# Antibacterial Efficiency and Osteoblast Viability of Ag/AgO/Ag_2_O Nanoparticles on Microarc-Oxidized TiO_2_

**DOI:** 10.1021/acsomega.5c09046

**Published:** 2026-01-23

**Authors:** Sitki Aktas, Salih Durdu, Toby W. Bird, Kadriye Ozcan, Gurkan Yigitturk, Salim Levent Aktug, Metin Usta, Tuba Acet, Andrew Pratt

**Affiliations:** † Department of Mechanical Engineering, Giresun University, Giresun 28200, Türkiye; ‡ School of Physics, Engineering and Technology, 8748 University of York, Heslington, York YO10 5DD, U.K.; § Department of Industrial Engineering, Giresun University, Giresun 28200, Türkiye; ∥ Department of Genetics and Bioengineering, Giresun University, Giresun 28200, Türkiye; ⊥ Department of Histology and Embryology, Mugla Sıtkı Kocman University, Mugla 48000, Türkiye; # Department of Materials Science and Engineering, 52962 Gebze Technical University, Gebze 41400, Kocaeli, Türkiye; ∇ Aluminum Research Center (GTU-AAUM), 52962 Gebze Technical University, Gebze 41400, Kocaeli, Türkiye; ○ Department of Occupational Health and Safety, Gumushane University, Gumushane 29100, Türkiye

## Abstract

Infections associated with titanium-based medical and dental implants present a major clinical challenge, as they can compromise osseointegration and long-term implant stability. Silver-based nanoparticles (NPs) are widely recognized for their strong antimicrobial properties, and when combined with titanium, they hold significant promise for developing infection-resistant and biocompatible implant surfaces. In this study, Ag/AgO/Ag_2_O NPs were deposited onto highly porous TiO_2_ layers formed on the Ti6Al4V alloy by microarc oxidation (MAO), with the aim of simultaneously enhancing antibacterial performance and supporting osteoblast activity. The NPs exhibited a predominant size of 8.7 ± 0.1 nm, with smaller particles oxidized to AgO and Ag_2_O, and larger particles (∼10 nm) composed of metallic Ag. SEM evaluation revealed that the NPs were homogeneously dispersed across the oxide surfaces without altering the rough and porous morphology of TiO_2_. The MAO-treated surfaces initially showed hydrophobic behavior (contact angle of 94.1 ± 0.3°), which shifted to hydrophilic after Ag/AgO/Ag_2_O NP deposition due to increased hydroxyl group formation. Antibacterial assays against *Escherichia coli* (*E. coli*) and *Staphylococcus aureus* (*S. aureus*) revealed a significant enhancement in antibacterial activity, particularly for surfaces with the highest Ag/AgO/Ag_2_O NP density. Meanwhile, osteoblast cell viability assays demonstrated no reduction in metabolic activity after 72 h, and SEM images confirmed cell adhesion and proliferation. Overall, these findings highlight the potential of Ag/AgO/Ag_2_O NP-modified TiO_2_ surfaces as multifunctional coatings that combine infection resistance with osteoblast compatibility, offering promising applications in dental and orthopedic implants.

## Graphical abstract

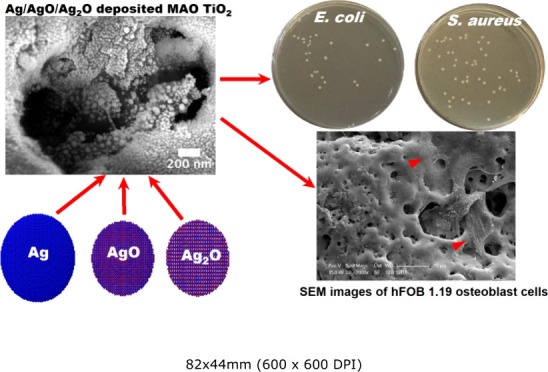

## 1 Introduction

Titanium and its alloys exhibit several advantageous properties that render them highly compatible with biological systems. These properties include high strength, excellent corrosion resistance, and the capacity to form a protective oxide layer of titanium dioxide on an implant. Consequently, these properties render titanium implants suitable for utilization in a wide range of surgical procedures and implant applications within the medical field.^1–3^ Titanium alloys exhibit a Young’s modulus that is analogous to that of bone, thereby rendering them a highly suitable material for use as bone replacements. A plethora of titanium alloys have been extensively utilized in clinical applications; however, Ti6Al4V stands out as a prominent example. It exhibits exceptional mechanical strength and high similarity in compression and tension to bones.^4–6^ Nevertheless, due to their bioinert nature, titanium alloys are incapable of undergoing a chemical bonding process with bone.^7^ Consequently, it is imperative that the surfaces undergo modification via various coating processes such as anodic oxidation,^8–10^ sol–gel^11^ and microarc oxidation.^12–14^

Microarc oxidation (MAO), alternatively referred to as plasma electrolytic oxidation, has recently attracted significant attention due to its capability to generate porous oxide layers. This facilitates the proliferation of bone cells within the pores, consequently fostering a robust mechanical connection between the implant and the surrounding tissue.^2,14,15^ It is well-known that implant-associated infections are the leading cause of revision surgery of orthopedic and dental implants.^16,17^ For instance, Sagnori et al.^18^ reported that ratio of early dental implant failure associated with postoperative infection was 21.34%. Similarly, Camps-Font et al.^19^ reported that the patient-based prevalence of postoperative infections after implant placement was 2.80%, and 65% of patients were surgically retreated due to antibiotic failure. In the light of the documented implant failures attributable to the formation of biofilms, there have been endeavors to mitigate the risk of bacterial activity on the MAO coating. These endeavors have involved the introduction of important antibacterial agents, such as Ag, Cu and Zn, with the objective of reducing the probability of bacterial proliferation.^20–24^

Silver nanoparticles (Ag NPs) have become a prevalent component in the field of biomaterials production due to their ability to exhibit broad-spectrum antibacterial properties, which renders them effective against a wide range of bacterial strains.^25–29^ The incorporation of Ag NPs into biomaterials has been demonstrated to exert a favorable effect against both Gram-positive and Gram-negative bacteria.^30–32^ The antibacterial mechanism of Ag NPs is well understood. It is known that Ag ions are released, which results in the degradation of the peptidoglycan component of the bacterial cell wall. This is followed by the inhibition of protein synthesis and binding to ribonucleic acid (RNA). Finally, the interaction with deoxyribonucleic acid (DNA) causes damage to replication signals, leading to the death of the bacterial cell.^33–35^

It is also well-known that antibacterial activity of NPs depends on their stability, size, shape, and surface chemistry which are controlled by synthesis methods.^36–40^ Electrophoretic deposition,^41^ chemical synthesis,^42^ calcination,^43^ atomic layer deposition,^44^ the spray deposition method^45^ electrochemical in situ deposition technique^46^ and gas aggregation method^47^ can be listed as deposition methods Ag NPs to enhance antibacterial performance of entire surfaces. Among these, the use of high-vacuum-compatible gas phase NP synthesis methods is preferable since it allows for the controlled production of NPs with specific shapes and a well-defined size distribution.^48–50^ Additionally, size effects of Cu@CuO core–shell NPs produced using gas aggregation cluster source on their antibacterial properties and cytotoxicity have been recently reported by our group.^51^

In this study, we aimed to investigate the effect of the concentration of gas-phase-produced Ag/AgO/Ag_2_O nanoparticles, which provide a multistage ion release mechanism for sustained efficacy, on their antibacterial activity and cytotoxicity. Initially, the surfaces of Ti6Al4V substrates were modified using the MAO method. The surface morphology and elemental composition were analyzed using scanning electron microscopy (SEM). The morphology and size distribution of NPs were examined via transmission electron microscopy (TEM). The binding energy and surface chemistry of nanoparticle-deposited surfaces were determined using X-ray photoelectron spectroscopy (XPS) and wettability was assessed using a contact angle goniometer. Finally, the effect of NP concentration on antibacterial activity against *Staphylococcus aureus* (*S. aureus*) and *Escherichia coli* (*E. coli*) which are the most common pathogens associated with orthopedic implant infections,^52,53^ as well as their cytotoxicity, was investigated.

## 2 Materials and Methods

Prior to the MAO process, rectangular Ti6Al4V (Grade-5) substrates were polished using sandpaper with grit sizes ranging from 180 to 1200. Following this process, the resulting substrates were cleaned in an ultrasonic bath and dried under warm air produced by a heat gun.

### 2.1 Microarc Oxidation (MAO)

The MAO apparatus comprises a 304 stainless steel receptacle and a system of agitation and refrigeration, operated by an alternating current power supply. The Ti6Al4V substrate and 304 stainless steel container were employed as the anode and cathode, respectively. The electrolyte was prepared with 10 g/L Na_3_PO_4_, 1 g/L KOH, and deionized water. MAO was conducted at 150 μF and 0.34 A/cm^2^ for 10 min at a temperature below 30 °C. Following the MAO step, all samples were ultrasonically cleaned in distilled water through 30 min in an ultrasonic bath and they were dried by a heat gun.

### 2.2 Nanoparticle Synthesis

Ag NPs were deposited onto the substrate using the NL50 gas aggregation cluster source from Nikalyte Ltd. A magnetron sputter head sits in a bullet shaped chamber and the flow of Ar is precisely controlled using mass flow controllers. Ar then sputters a target material which creates a supersaturated vapor which aggregates out to form NPs.^54^ Differential pumping between the aggregation chamber and the sample chamber forms a large pressure gradient creating a jet-like expansion of the gas which carries the particles along. This forms a beam of NPs which then deposit on the sample surface with the size of the particles dictated by the Ar gas flow rate and the plasma power. Typical operating conditions for the growth of the Ag particles was 40 sccm of Ar and a plasma power of 50 W. Ag-based NPs were subsequently deposited onto the modified surfaces at three different concentrations, with deposition times of 4, 8, and 12 min. The resulting samples are herein designed as MAO-Ag1, MAO-Ag2 and MAO-Ag3, respectively.

### 2.3 Surface Characterization

The morphology, size, and size distribution of the Ag NPs produced using the NL50 nanoparticle deposition system on a TEM grid were evaluated via transmission electron microscopy (TEM, JEOL 2100+). Scanning electron microscopy (SEM, JEOL 7800F Prime) and SEM-energy-dispersive X-ray (SEM-EDX)-area and -mapping (SEM, Hitachi SU1510) were conducted to investigate the morphology, composition and elemental distribution of the surfaces, respectively.

Binding energies were attributed using the NIST database.^55^ The binding energies and the surface chemistry of the surfaces were evaluated with Al Kα radiation (1486.61 eV) by monochromated X-ray photoelectron spectroscopy (XPS, Omicron EA125). Peak deconvolution and fitting were carried out manually using the CasaXPS software.

A sessile drop technique was employed to measure the average contact angle of the surfaces. A 1 μL water droplet was placed on the surfaces for 1 min, and the contact angle was measured using a contact angle goniometer (Dataphysics OCA-15EC). The surface free energy (SFE) of the surfaces was calculated using the average contact angle values by applying the Neumann method. This method is based on an assumption between SFE of a solid, SFE of a wetting liquid and SFE of a solid–liquid interface.^56^

### 2.4 Microbial Adhesion Test

Microbial adhesion experiments were conducted with *S. aureus* and *E. coli*. Prior to the commencement of the experiment, all samples were sterilized in an autoclave. Subsequently, the test microorganisms were adjusted in accordance with the 0.5 McFarland scale and treated with the bare Ti6Al4V (control), bare MAO and Ag NP-coated MAO samples. For this process, samples measuring 1 cm^2^ were immersed in 5 mL of MHB medium. Following a 24-h incubation period at 37 °C with 125 rpm agitation on an orbital shaker, the samples were removed from the medium and washed with 15 mL of water to remove nonadherent organisms. This process was repeated three times before each sample was placed in a clean tube and 2 mL of 150 mM NaCl was added and vortexed for 2 min to collect the bacteria attached to the sample surface. Serial dilutions of the obtained bacterial solution were prepared, and 100 μL was taken from each dilution and applied to the MHA medium by the spreading method. Following a 48-h incubation period at 37 °C, a colony count was conducted, and the percentage inhibition was calculated using the following formula:

$$\mathrm{Inhibition}\%=\frac{{\mathrm{BCC}}_{\mathrm{uncoated}}-{\mathrm{BCC}}_{\mathrm{coated}}}{{\mathrm{BCC}}_{\mathrm{uncoated}}}\times 100$$ 1

where BCC is the known bacterial colony count for each sample.

### 2.5 WST-8 Cell Viability Analysis

hFOB 1.19 osteoblast cells were seeded on samples in 24-well culture dishes at 5 × 10^4^/well/200 μL. Osteoblast cells were cultured for 24 h in Ham’s F12 Medium Dulbecco’s Modified Eagle’s Medium (1:1), with 2.5 mM l-glutamine at 37 °C, 5% CO_2_ and 90% humidity conditions. For measurement, 20 μL of WST-8 solution was added to each well and incubated for 3 h at 37 °C. After incubation, 100 μL of medium was taken from each well of the 96-well culture dish and absorbance measurements were performed at a wavelength of 450 nm.

### 2.6 SEM Analysis

hFOB 1.19 osteoblast cells were seeded on samples in 24-well culture dishes at 5 × 10^5^/well/1000 μL. After the experiment was completed, the cell-coated samples were fixed with 2.5% glutaraldehyde (v:v, 0.1 M pH 7.4 PBS) for 1 h at room temperature and washed with 0.1 M pH 7.4 PBS. Cells were dehydrated by passing through an ethanol series with increasing concentrations (50, 70, 90, and 96%). The samples were air-dried, coated with Au and examined under a scanning electron microscope.

### 2.7 Statistical Analysis

All analyses were realized in five replicates. Variance analysis of the mean values was performed with the Duncan Multiple Comparison test (one-way ANOVA) using SPSS software for Microsoft Windows (Ver. 20.0, SPSS Inc., USA) and the significance level was determined at the 5% level (*p* < 0.05).

## 3 Results and Discussion

Ag NPs were deposited using the NL50 deposition system onto a TEM grid under the same conditions as for the MAO surface deposition, but for a shorter time, to investigate their size, shape, and composition. Figure 1a shows low magnification TEM images of Ag NPs, which are mostly spherical and exhibit a range of sizes. Figure 1b presents the size distribution of Ag NPs, evaluated from TEM images using ImageJ. The data indicate that the NPs follow a log-normal size distribution, with the most probable size being 8.8 ± 0.3 nm. In addition, the size distribution of Ag NPs was determined from two different regions of the TEM grid, with the most probable sizes calculated from each image being 8.0 ± 0.2 and 8.8 ± 0.1 nm, respectively (Figure S1). Hence, the average of three size distributions indicates that the most probable size of Ag NPS is 8.7 ± 0.1 nm. It is also clear that there are smaller NPs below 2 nm in size, but these have not been included due to limitations in measurement and calculation accuracy. Additionally, it is apparent that while the smaller NPs are fully oxidized, the larger ones (∼10 nm diameter), which appear darker in Figure 1a, remain in the form of pure Ag. It is well-known that the oxidation of NPs is highly dependent on their size and shape.^57^

**Figure 1:**
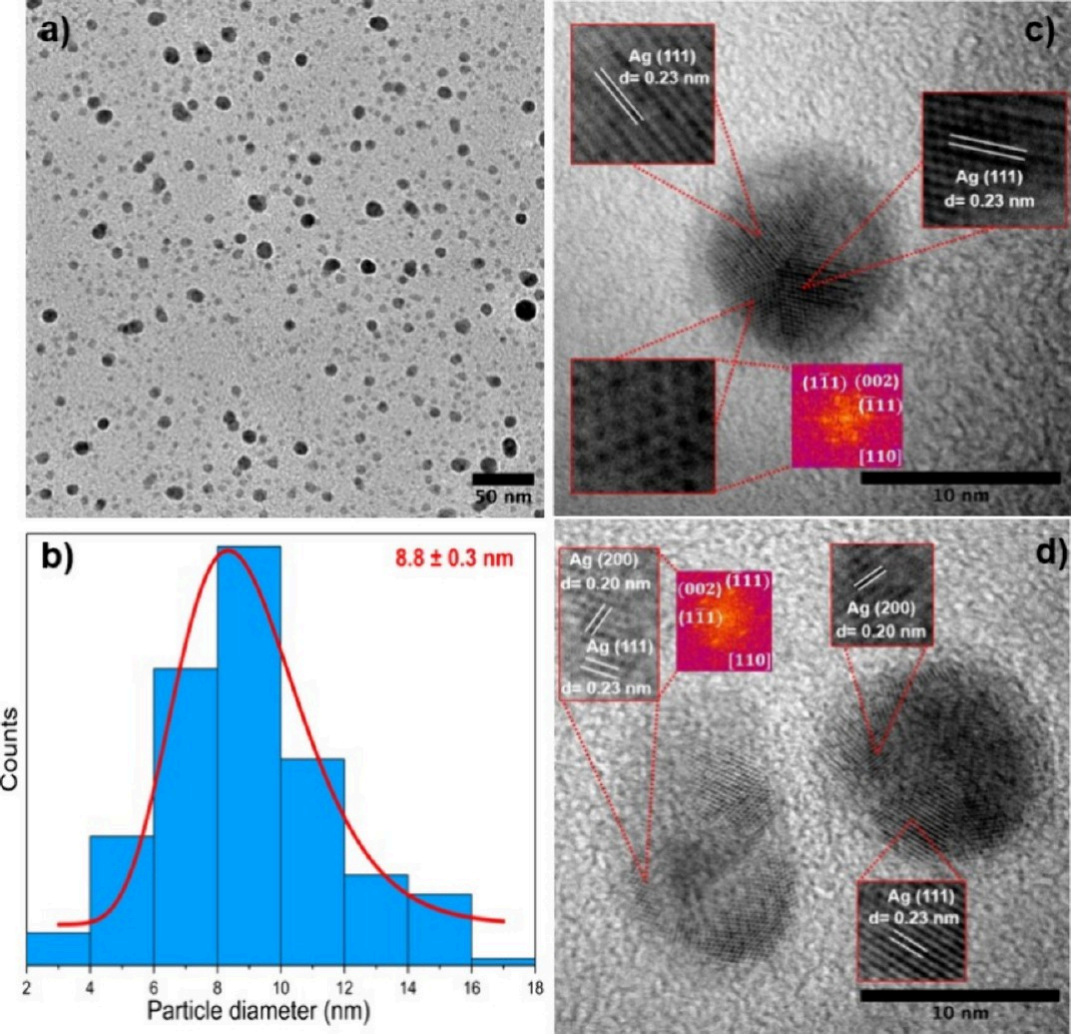
(a) Low magnification TEM image of Ag NPs deposited on a TEM grid. (b) Size distribution of Ag NPs determined from the TEM image shown in (a). (c,d) show high magnification images of two different Ag NPs. Zoomed areas are shown as inset images with *d*-spacing values and Fast Fourier Transform (FFT) of one zoomed area in each image. The findings clearly show that larger particles are pure Ag.

Further investigation was carried out using high magnification TEM images of different NPs, approximately 10 nm in diameter, revealing several crystalline planes. As illustrated in Figure 1c, different regions of the NPs were investigated, and interplanar distances were determined using FFTs (Fast Fourier Transforms) of each region. One of the FFT images is provided as an inset, indicating the (11̅1) and (002) planes of Ag (FCC) along the [110] zone axis orientation. Additionally, interplanar distances were measured to be 0.23 nm, corresponding to the (111) crystalline plane of metallic Ag. As shown in Figure 1d, similar features were observed in other NPs, with the (200) crystalline plane also detected, having an interplanar distance of 0.20 nm, which also corresponds to metallic Ag.

Top-view SEM images of Ag NPs deposited on MAO-treated surfaces are presented in Figure 2. The rough and porous surface morphology, which is beneficial for surface bioactivity, resulting from the MAO process on oxide surfaces presented in Figure S2, remains present across all levels of Ag NP deposition, from lower to higher amounts, as shown in the low-magnification SEM images (Figure 1a,d,g, respectively). SEM images obtained at higher magnifications indicate that the Ag NPs are homogeneously distributed on the surfaces, with the concentration of NPs increasing with deposition time. Additionally, the pore structures’ surfaces are also decorated with Ag NPs, which may enhance the antibacterial activity across the entire active surface.

**Figure 2:**
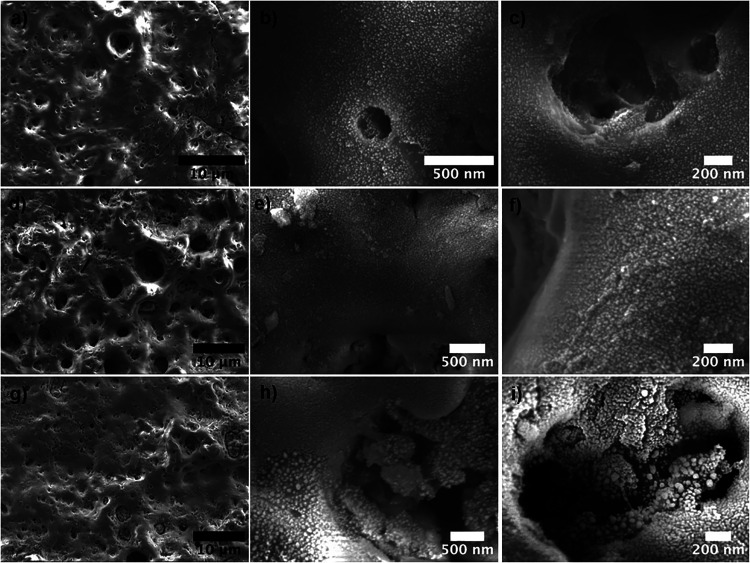
Top view SEM images of Ag NPs deposited on MAO surfaces. (a–c) MAO-Ag1, (d–f) MAO-Ag2, and (g–i) MAO-Ag3 from low to high magnifications.

The elemental composition of bare Ti, MAO surfaces, and Ag-deposited MAO surfaces was investigated both quantitatively and qualitatively using EDX-area and EDX-mapping analysis. It is evident that bare Ti possesses the composition of Ti6Al4V, with Ti (wt % = 90.6), Al (wt % = 6.7), and O (wt % = 3.8). Furthermore, as a result of the MAO process, a significant amount of O (wt % = 46.8) was detected on the MAO surface, along with additional P and Na elements, as listed in Table S1. The presence of P and Na is attributed to the Na_3_PO_4_-based MAO solution used during the oxidation process of bare Ti. Moreover, Ag-deposited MAO surfaces showed the presence of all the aforementioned elements, with the amount of Ag varying depending on the deposition time, as shown in Table 1. For instance, the MAO-Ag1 surface, which underwent the shortest deposition time, contained 0.5 wt % Ag, while the surfaces subjected to the medium and longest deposition times showed 1.1 and 1.5 wt % Ag, respectively. These results indicate that the amount of deposited Ag increased with longer deposition times, with the MAO-Ag2 and MAO-Ag3 samples containing approximately two and three times more Ag compared to the MAO-Ag1 sample. Additionally, EDX-mapping images of the MAO-Ag1, MAO-Ag2, and MAO-Ag3 samples are presented in Figure 3. It is evident that Ti, O, and Ag are homogeneously distributed across all surfaces, and the increase in Ag content with deposition time is clearly detectable.

**Table 1:** EDX Area Analysis of MAO Surfaces Deposited with Three Different Surface Densities of Ag-Based NPs

map sum spectrum	MAO-Ag1			MAO-Ag2			MAO-Ag3		
element	wt %	**σ**	at. %	wt %	**σ**	at. %	wt %	**σ**	at. %
**O**	45.5	0.5	69.7	45.4	0.5	69.7	43.8	0.5	68.5
**P**	5.6	0.1	4.5	6.1	0.1	4.8	5.5	0.1	4.4
**Ti**	45.1	0.5	23.1	44.4	0.4	22.7	46.2	0.4	24.1
**Al**	1.9	0.1	1.7	1.8	0.1	1.7	1.7	0.1	1.6
**V**	0.8	0.2	0.4	0.7	0.2	0.4	0.9	0.2	0.4
**Na**	0.6	0.1	0.6	0.5	0.1	0.6	0.5	0.1	0.6
**Ag**	0.5	0.1	0.1	1.1	0.1	0.2	1.5	0.2	0.3
**total**	100		100	100		100	100		100

**Figure 3:**
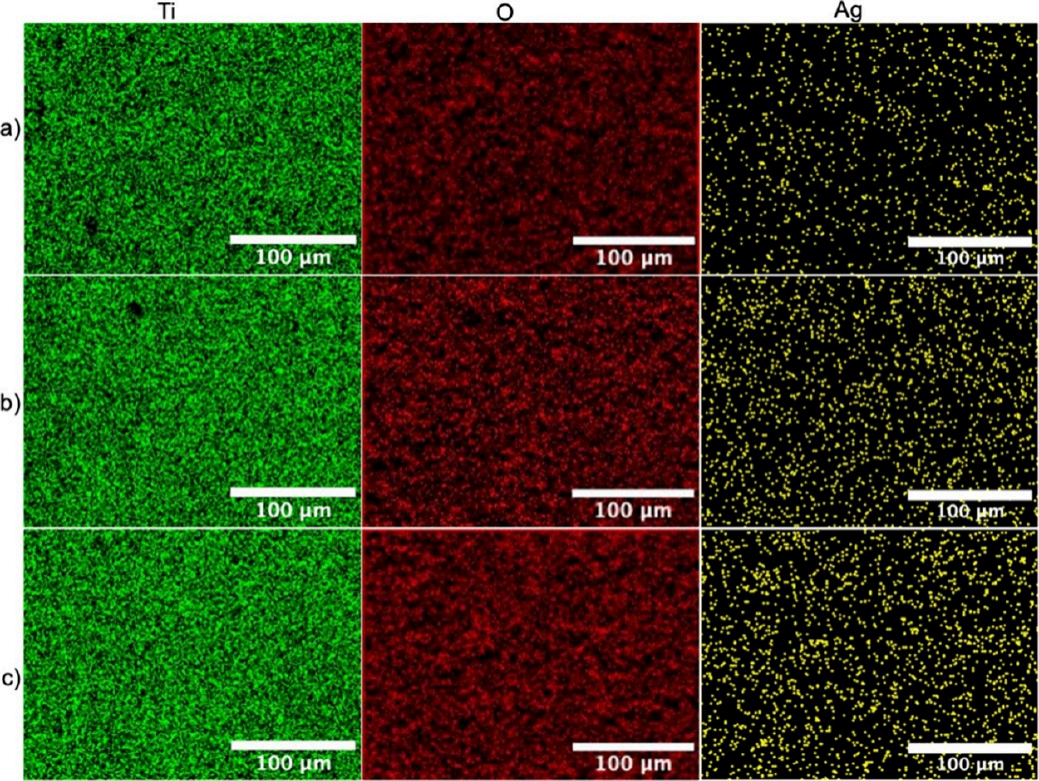
EDX mapping images (green: Ti, red: O, and yellow: Ag) of Ag NP-deposited MAO surfaces, arranged from low to high surface density: (a) MAO-Ag1, (b) MAO-Ag2, and (c) MAO-Ag3.

XPS analysis in Figure 4 shows that there are large levels of oxidation across the three samples, especially in MAO-Ag1 and MAO-Ag2. The oxide signal most likely originates from the fully oxidized particles seen in the TEM images. The stronger Ag metal signal in MAO-Ag3 is probably due to the fact that the higher deposited amount of Ag has resulted in aggregated NPs on the Ti surface meaning less oxidation of the NPs in the sublayers. The relative signals of the C and O also decreased as the deposition time increased, giving further evidence that more NPs were deposited due to the longer deposition time.

**Figure 4:**
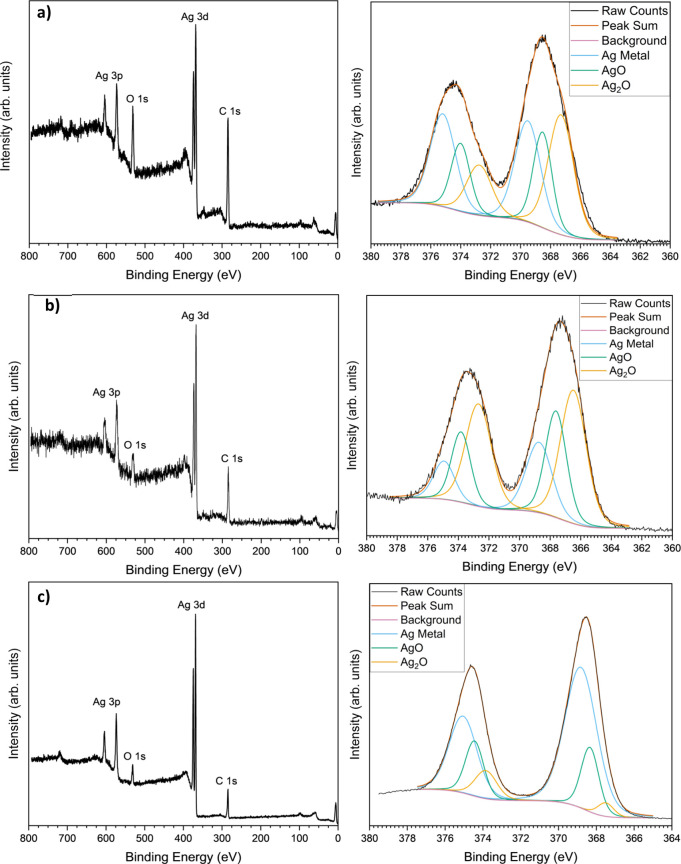
XPS spectra of Ag NP-deposited MAO surfaces. (a) MAO-Ag1, (b) MAO-Ag2, and (c) MAO-Ag3. The left side shows the survey scan, while the right side displays the high-resolution Ag 3d peaks.

### 3.1 Wettability Test

The average contact angle and SFE of all samples are illustrated in Figure 5. The measurement of contact angles is a method used to ascertain the wettability of a given surface, which can be categorized as either hydrophilic or hydrophobic. In the event of the contact angle value being less than 90°, the surface is observed to exhibit hydrophilic properties. Conversely, should the value exceed 90°, hydrophobic properties are exhibited.^58^ A lower contact angle is indicative of a greater SFE and hydrophilicity, whereas a larger contact angle is associated with a lower SFE and hydrophobicity. The average contact angle of the Ti6Al4 V substrates was measured to be 61.7 ± 0.1°, indicating a hydrophilic nature. The average contact angle of the MAO surface was 94.1 ± 0.3°, indicating a hydrophobic nature. The average contact angle of the MAO-Ag1, MAO-Ag2, and MAO-Ag3 surfaces were 76.2 ± 0.2°, 77.1 ± 0.3°, and 83.2 ± 1.6°, respectively, showing hydrophilic behavior. The findings illustrate that the uncoated MAO surface displays enhanced wettability in comparison to the substrate surface. The water droplet initially contacts the protective passive TiO_2_ oxide layer on the substrate surface. The layer in question is naturally present on the substrate surface, and the surface exhibits hydrophilic characteristics due to its partially hydroxylated polar structure. However, following contact between the droplet and the MAO surface, the atmospheric gases within the tubes create a temporary resistance, preventing the wettability of the surface. This results in higher contact angles, indicating that the MAO surface exhibits hydrophobic behavior in comparison to the substrate surface. Furthermore, Ag NPs-deposited MAO surfaces display a reduced contact angle in comparison to the MAO samples. Ag possesses high oxygen affinity and is easily oxidized under atmospheric conditions as verified by the existence of AgO and Ag_2_O in the XPS spectra. This contributes to increasing the amount of hydroxyl groups on the surface. It can be postulated that the presence of augmented hydroxyl groups on Ag NPs-deposited MAO surfaces may lead to an enhancement in wettability.

**Figure 5:**
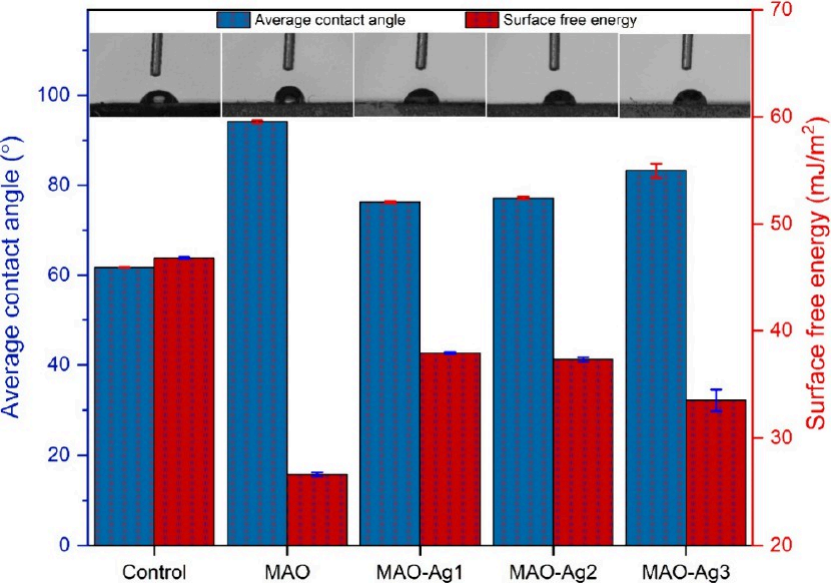
Average contact angle and surface free energy (SFE) values of all surfaces, as determined by a sessile constant drop technique within 1 min after 1 μL water droplets contacted the surfaces.

### 3.2 Microbial Tests

Antibacterial activity was assessed by colony counting of bacteria adhering to the surfaces as given in Table 2 and Figures 6 and S3. In the colony counting test, a low number of colonies obtained from the surface indicates a high level of antibacterial activity. It was established that the antibacterial efficacy of MAO surfaces produced through MAO application on control surfaces, demonstrated an enhancement. It is hypothesized that the observed efficacy is due to the inactivation of bacteria within the gaps that are formed on the surface. The antibacterial activity of surfaces formed by the addition of varying quantities of Ag/AgO/Ag_2_O NPs to MAO surfaces was observed to increase in proportion to the quantity of Ag/AgO/Ag_2_O present.

**Table 2:** Microbial Adhesion to the Surfaces (CFU 10^2^/mL)^a^

samples	***S. aureus***	**inhibition (%)**	***E. coli***	**inhibition (%)**
**control**	206 ± 4^a^		115 ± 2^c^	
**MAO**	148 ± 2^b^	28.2	92 ± 3^d^	20
**MAO-Ag1**	118 ± 1^c^	42.7	73 ± 1^e^	36.5
**MAO-Ag2**	67 ± 2^f^	67.5	52 ± 2^g^	54.8
**MAO-Ag3**	48 ± 2^h^	76.7	31 ± 2^i^	73.0

a ± means ± SD of three replicates. Lowercase letters ^(a‑i)^ indicate statistically significant differences according to Duncan’s multiple range test (*P* < 0.05).

**Figure 6:**
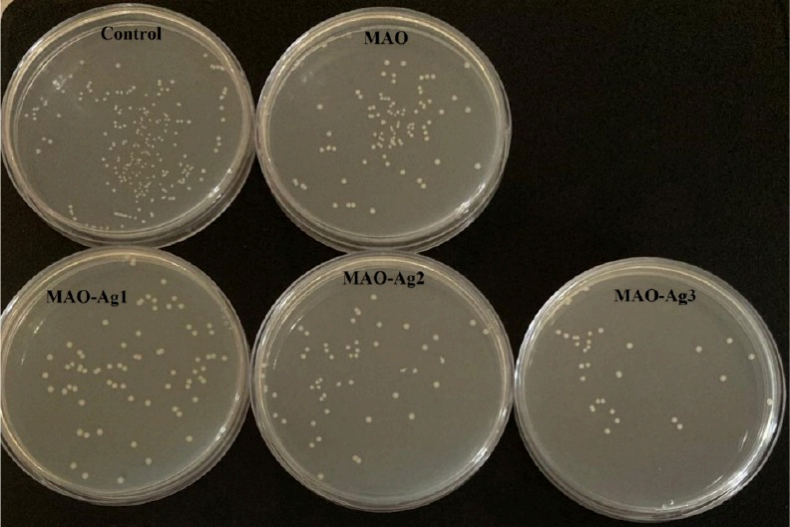
Reduction in microbial colonies after reculturation in the Petri dishes for *E. coli*.

It is well-known that the antibacterial activity of a surface is contingent on various factors, including but not limited to surface chemistry, wettability, roughness, and texture. As the contact angle is increased, the surface undergoes a transformation from a hydrophilic structure to a hydrophobic structure. Consequently, the probability of water molecules encountering the surface is reduced. Thus, bacterial adhesion becomes more difficult onto a hydrophobic surface. This is advantageous in terms of corrosion prevention and antibacterial properties. According to the wettability results, the wettability included from hydrophilicity to hydrophobicity had decreased with increasing Ag/AgO/Ag_2_O NP. This directly affected antibacterial activity, and bacterial adhesion was reduced with increasing Ag/AgO/Ag_2_O NP. Another important parameter on antibacterial activity is surface chemistry. The Ag-modified surface chemistry significantly enhanced the antibacterial response by promoting higher Ag^+^ ion release. Ag^+^ ions are well-known to interact with thiol-containing membrane proteins, disrupting membrane integrity, inhibiting respiratory chain enzymes, binding to bacterial nucleic acids, and collectively disrupting fundamental cellular functions.^59^ Therefore, the antibacterial activity of Ag-containing MAO coatings stems from the synergistic effect of potent Ag^+^-mediated bactericidal mechanisms, including reduced bacterial adhesion and membrane dysfunction due to altered wettability, metabolic inhibition, and DNA damage.

The antibacterial activity of Ag NPs has been attributed to the dissolution of Ag NPs, the release of Ag^+^ ions affecting cell membrane permeability, and direct particle–cell wall interactions.^60,61^ Xiu et al.^62^ reported that the antibacterial activity of Ag NPs can be regulated by modulating the release of Ag^+^ ions, potentially through the manipulation of oxygen availability. Taglietti et al.^63^ studied antibacterial activity of glutathione-coated Ag NPs and highlighted that two factors which play important role in antibacterial mechanism. These are the release of Ag^+^ ions into the exposure medium, described as a “long-distance mechanism,” and the interactions occurring at the particle–bacterial membrane interface, referred to as a “short-distance mechanism. Additionally, Lok et al.^64^ reported that while zero-valent Ag does not exhibit antibacterial activity, oxidized Ag NPs demonstrate significant antibacterial properties. Similarly, Rebelo et al.^65^ observed comparable behaviour in Ag NPs through their evaluation of the antibacterial activity of magnetron-sputtered Ag and AgO_*x*_ thin films. They identified AgO_*x*_ as the primary contributor to the antibacterial effect, attributing it to increased production of reactive oxygen species (ROS). Furthermore, the antibacterial activities of AgO and Ag_2_O NPs have also been investigated, with studies highlighting their potential as promising candidates.^66,67^ Bonilla-Gameros et al.^68^ stated that AgO has fast continuous release of Ag^+^ release compared to Ag, resulting better antibacterial activity. Additionally, it has been reported that smaller Ag NPs exhibit higher antimicrobial activity compared to larger ones.^69^ Morones et al.^70^ used scanning transmission electron microscopy (STEM) to identify the location and distribution of Ag NPs on bacterial surfaces. Their findings revealed that only Ag NPs with a size range of 1–10 nm could bind to bacterial membranes. Moreover, they observed that these membrane-bound NPs were able to penetrate the bacteria. The higher efficiency of smaller Ag NPs is attributed to their larger surface-to-volume ratio, which enhances their direct interaction with bacterial cells, ultimately leading to cell death. As indicated by the XPS discussion and TEM image analyses, while metallic Ag NPs are present, AgO and Ag_2_O phases are also observed. The size of the NPs ranges from 1 to 16 nm, with the majority falling between 1 and 12 nm and a most probable size of 8.7 ± 0.1 nm. It is difficult to separate the specific contributions of Ag, AgO, and AgO_2_ nanoparticles, as all coated surfaces contained the same composite nanoparticles at different concentrations. Nevertheless, based on both literature and the present findings, the antibacterial activity of Ag/AgO/Ag_2_O NPs can be attributed to multiple mechanisms. These include direct contact between silver/silver oxide and bacterial cells, leading to membrane deformation, as well as interactions with essential intracellular molecules that impair cellular function. In addition, the multi-stage ion release from Ag/AgO/AgO_2_ nanoparticles produces Ag^+^ ions, which disrupt the bacterial outer membrane, enhance the internalization of both nanoparticles and ions, and ultimately reduce cell viability. In this study, MAO-Ag1 and MAO-Ag2 showed approximately 40% and 60% inhibition against *E. coli* and *S. aureus*, respectively, while MAO-Ag3 exhibited the highest antibacterial activity with around 75%, correlating with its higher deposition density. Although the antibacterial activity of approximately 75% is below the FDA’s expected threshold for qualifying as an active antimicrobial biomedical device,^71^ this work represents an important initial investigation; moreover, optimization of nanoparticle size and shape may further enhance its antibacterial performance in future studies.

### 3.3 hFOB 1.19 Osteoblast Cell Viability

In viability analyses, the highest optical density (OD) value was detected in MAO-Ag2 at 72 h and the lowest OD value was detected in the control at 24 h. No statistically significant differences were found between control, MAO-Ag2, and MAO-Ag3 at 24 h. However, MAO and MAO-Ag1 were found to be significantly higher compared to the control (*p* < 0.05). At 48 h, MAO was found to be significantly higher compared to other samples (*p* < 0.05). No significant differences were observed between the OD values at 72 h. OD values and standard errors of the samples are presented in Table S2 and graphed in Figure 7.

**Figure 7:**
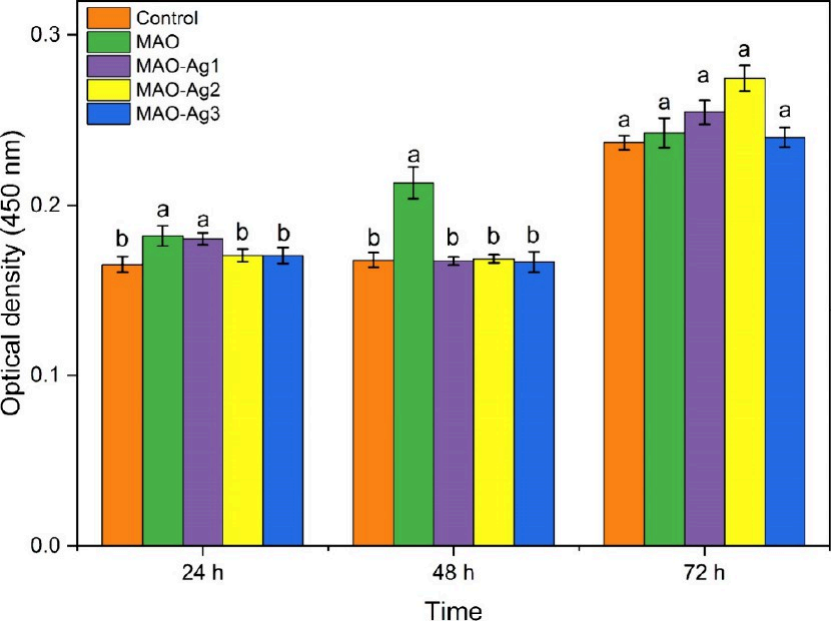
Optical density (OD) values and standard deviations of the viability analysis at 24, 48, and 72 h for the samples shown in the legend. Lowercase letters indicate statistically significant differences according to Duncan’s multiple range test (*P* < 0.05).

### 3.4 SEM Imaging

Scanning electron microscope photographs showed that osteoblast cells were attached to the samples (Figure 8). Examining the quantity of filopodia and lamellipodia, no statistically significant difference was found between the samples.

**Figure 8:**
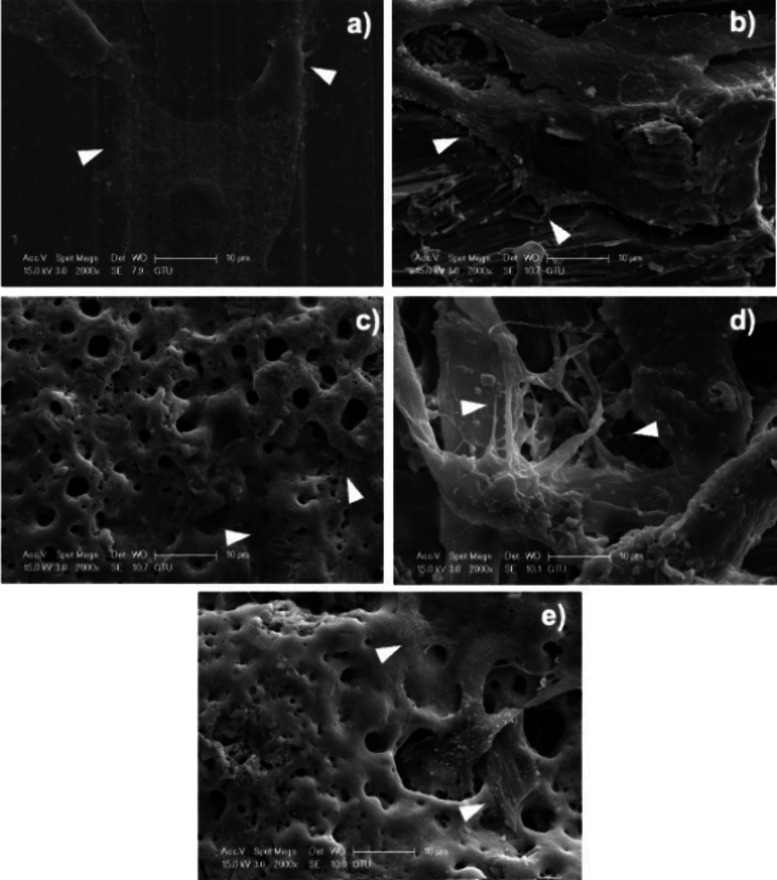
SEM micrographs of hFOB 1.19 osteoblast cells. (a) control, (b) MAO, (c) MAO-Ag1, (d) MAO-Ag2, and (e) MAO-Ag3. Arrowhead: Filopodia and lamellipodia. Magnification 2000×. Scale bars indicate 10 μm.

Although cell proliferation appeared to be higher in the MAO-Ag1 group at the 24 h, quantitative analysis at 72 h indicated no significant difference in the number of cells adhered to the material surfaces between the groups. This suggests that the initial proliferative response observed may not have translated into sustained adhesion over time. SEM images in Figure 8 provided visual confirmation of osteoblast attachment on all material surfaces, with cells exhibiting typical spreading morphology, cytoplasmic extensions, and close interaction with the substrate. These morphological indicators support the notion that the tested materials, including MAO-Ag1, possess favorable surface characteristics that support osteoblast proliferation and adhesion. The unchanged number of adhered cells at 72 h may indicate that the surface became fully covered, leaving no area for more cells to attach, or that cell growth and detachment reached a balance. These results show the importance of studying how cells behave on implant surfaces over time to understand how safe and effective the material is for supporting bone healing While these models provide valuable insights, future clinical applications must consider biofilm formation and antibiotic-resistant strains. As this study assessed implant toxicity only in vitro, further animal and clinical studies are needed to evaluate potential systemic toxicity. Moreover, future work should include testing against additional bacterial species, including drug-resistant pathogens and polymicrobial biofilms, to better reflect clinical scenarios.

## 4 Conclusions

Ag NPs were deposited in the gas-phase at three different densities onto TiO_2_-based surfaces produced on Ti6Al4V substrates using the MAO method. The XPS analysis revealed that the NPs formed as metallic Ag, AgO, or Ag_2_O, depending on their size. TEM images confirmed that most of the NPs existed in oxide form. Furthermore, the porous and rough morphology of the MAO surfaces remained after the deposition of Ag NPs at all concentrations, and Ti, O, and Ag were homogeneously distributed across the sample surfaces. Additionally, the deposition of Ag/AgO/Ag_2_O NPs transformed the originally hydrophobic MAO surface into one exhibiting hydrophilic property. Antibacterial activity tests demonstrated that the deposition of Ag/AgO/Ag_2_O NPs significantly enhanced the antibacterial performance of the MAO surface against *E. coli* and *S. aureus*. The inhibition rate was found to be highest for MAO surfaces with the highest density of Ag/AgO/Ag_2_O NPs. Cell viability and SEM images indicated that osteoblast cells adhered to all surfaces and were able to proliferate. The findings of this study demonstrate that MAO-treated surfaces functionalized with Ag/AgO/Ag_2_O NPs serve as effective multifunctional coatings by significantly enhancing antibacterial performance while preserving cytocompatibility. This dual functionality highlights their potential as a promising approach for the surface engineering of advanced biomedical implants. Although the in vitro findings demonstrate promising antibacterial activity and cytocompatibility of the Ag/AgO/Ag_2_O NPs coating, further in vivo investigations, such as animal model studies, are essential to confirm its long-term safety, stability, and biological performance under physiological conditions.

## Supplementary Material



## Data Availability

The data supporting this article have been included as part of the Supporting Information.

## Notes

TEM images of Ag NPs deposited from two different regions of grid; histograms of size distribution of Ag NPs calculated corresponding TEM images (Figure S1); SEM image of MAO surface (Figure S2); EDX area analysis of Ti6Al4V and MAO surfaces (Table S1); reduction in microbial colonies after reculturation in the Petri dishes for *S. aureus* (Figure S3); OD values and standard deviations from the viability analysis at 24, 48, and 72 h for the control (Ti6Al4 V), MAO, and MAO surfaces deposited with three different densities of Ag/AgO/Ag_2_O NPs (Table S2) (PDF)
