# Supplementary material for: Antibacterial Efficiency and Osteoblast Viability of Ag/AgO/Ag2O Nanoparticles on Microarc-Oxidized TiO2
Source: ACS Omega. 2026 Jan 23;11(4):5526–37. doi: 10.1021/acsomega.5c09046 (PMC13105259; doi:10.1021/acsomega.5c09046)
Supplement: Supplementary file 1 [file ao5c09046_si_001.pdf]

Supplementary information

Antibacterial Efficiency and Osteoblast Viability of Ag/AgO/Ag_2O Nanoparticles on Micro-Arc Oxidized TiO_2

Sitki Aktas[a,b*], Salih Durdu[a,c*], Toby W. Bird[b], Kadriye Ozcan[d], Gurkan Yigitturk[e], Salim Levent Aktug[f], Metin Usta[f,g], Tuba Acet[h] and Andrew Pratt[b*]

[a] *Department of Mechanical Engineering, Giresun University, Giresun, 28200, Turkey*

[b] *School of Physics, Engineering and Technology, University of York, Heslington, York, YO10 5DD, United Kingdom.*

[c] *Department of Industrial Engineering, Giresun University, Giresun, 28200, Turkey*

[d] *Department of Genetics and Bioengineering, Giresun University, Giresun, 28200, Turkey*

[e] *Department of Histology and Embryology, Mugla Sıtkı Kocman University, Mugla, 48000, Turkey*

[f] *Department of Materials Science and Engineering, Gebze Technical University, Gebze, Kocaeli, 41400, Turkey*

[g] *Aluminum Research Center (GTU-AAUM), Gebze Technical University, Gebze, Kocaeli, 41400, Turkey*

[h] *Department of Occupational Health and Safety, Gumushane University, Gumushane, 29100, Turkey*

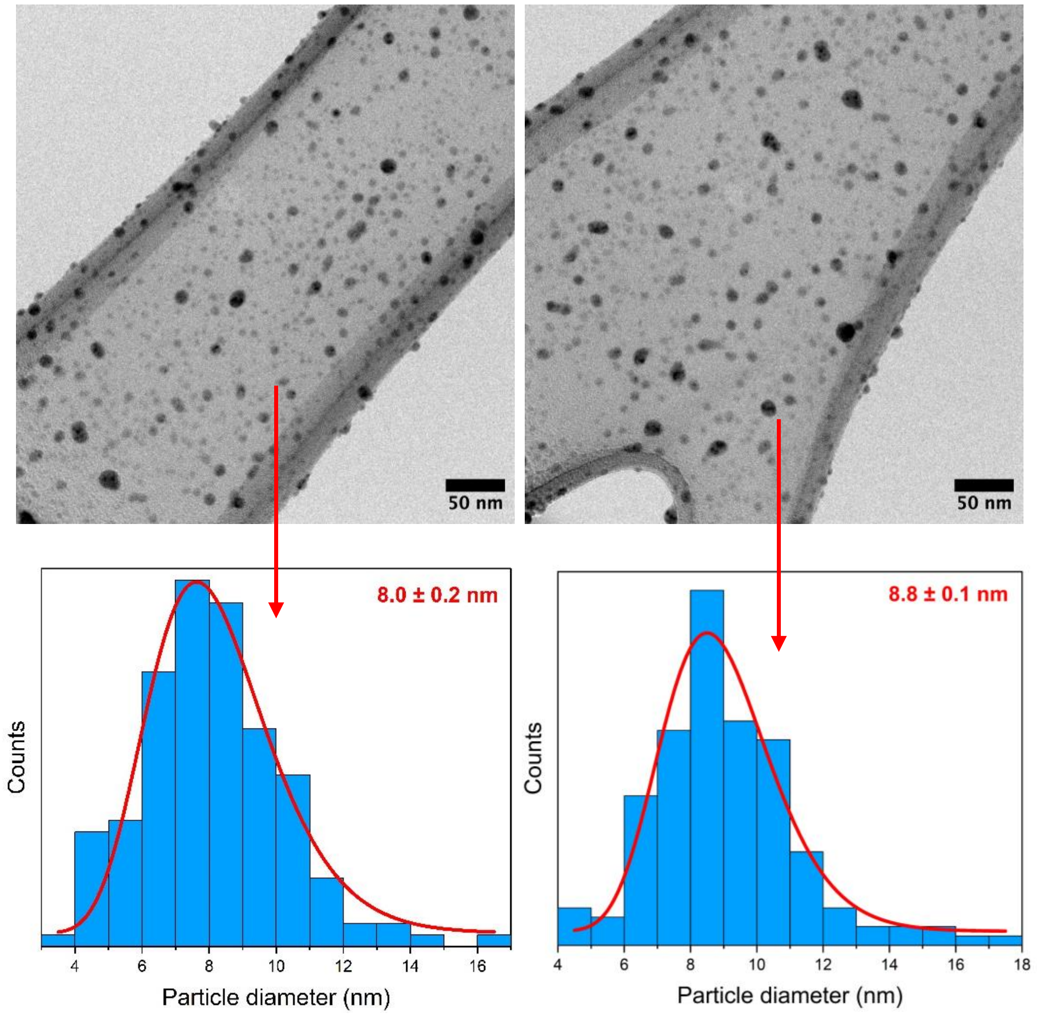

Figure S1: TEM images of Ag NPs deposited taken from two different regions of grid. Histograms of size distribution of Ag NPs calculated corresponding TEM images.

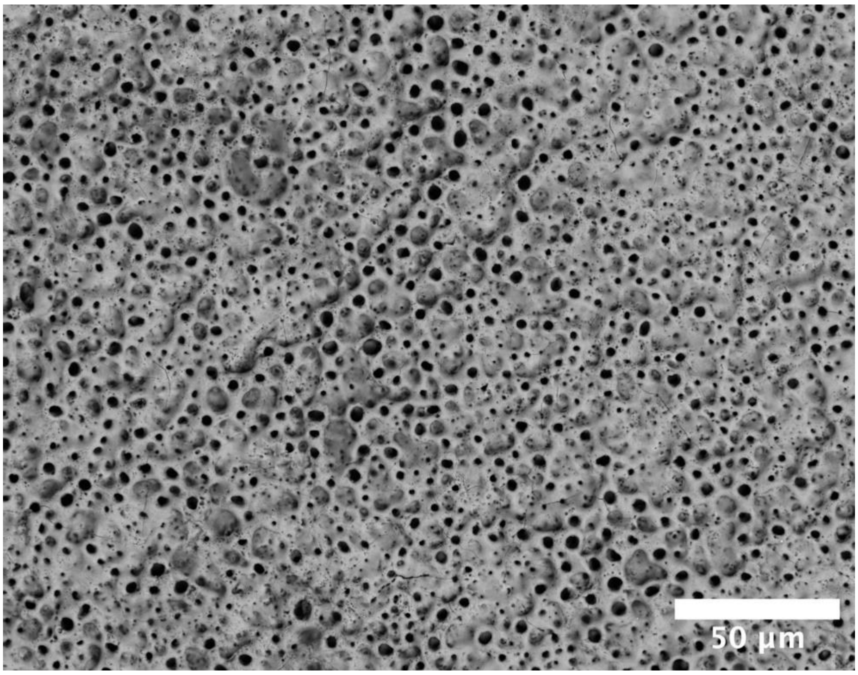

Figure S2: SEM image of MAO surface.

Table S1: EDX area analysis of Ti6Al4V and MAO surfaces.

Map Sum Spectrum	Ti6Al4V			MAO		
**Element**	**wt. %**	**σ**	**at. %**	**wt. %**	**σ**	**at. %**
**Ti**	**90.6**	**0.3**	**87.1**	**43.0**	**0.2**	**21.6**
**O**	**--------**		**--------**	**46.8**	**0.2**	**70.3**
**Al**	**5.6**	**0.1**	**9.5**	**2.1**	**0.0**	**1.9**
**V**	**3.8**	**0.3**	**3.5**	**0.9**	**0.1**	**0.4**
**P**	**--------**		**--------**	**6.6**	**0.1**	**5.1**
**Na**	**--------**		**--------**	**0.6**	**0.0**	**0.9**
**Total**	**100**		**100**	**100**		**100**

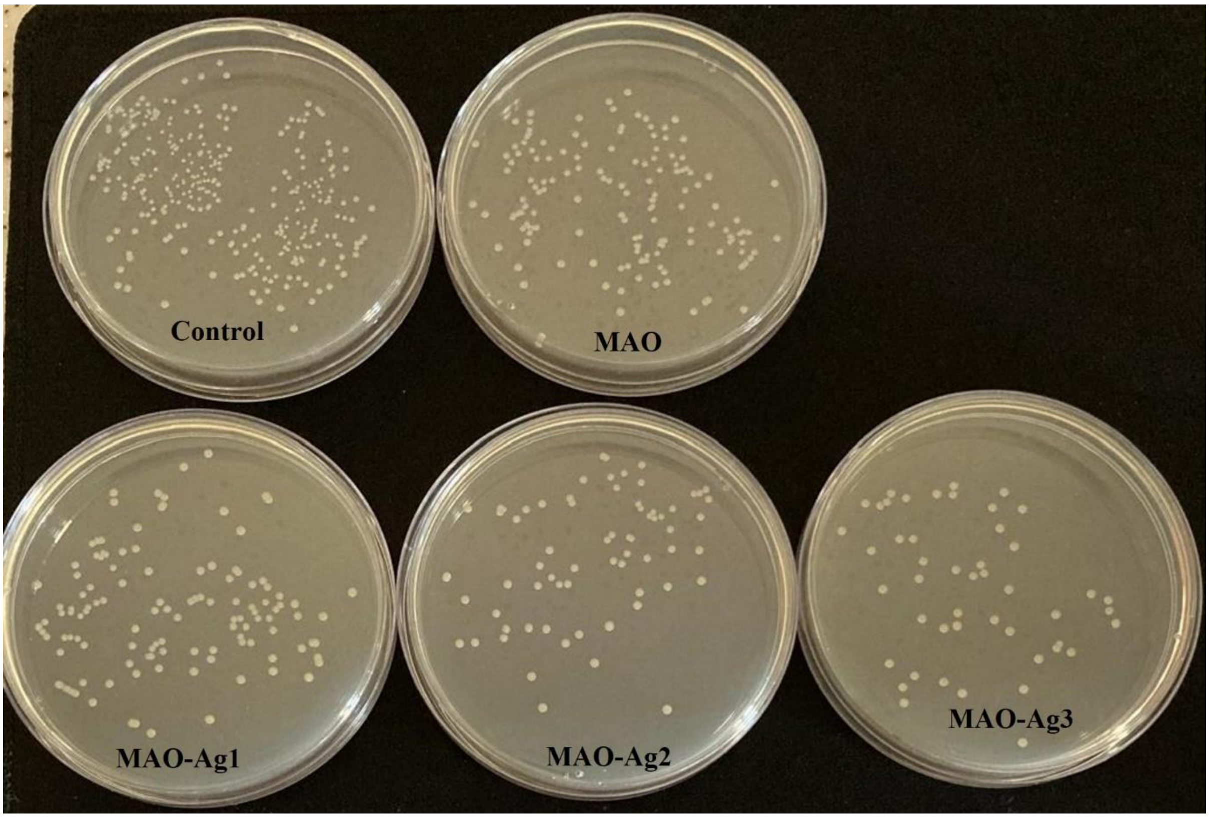

Figure S3: Reduction in microbial colonies after re-culturation in the petri dishes for *S. aureus*.

Table S2: Optical density (OD) values and standard deviations from the viability analysis at 24, 48, and 72 hours for the control (Ti6Al4V), MAO, and MAO surfaces deposited with three different densities of Ag/AgO/Ag_2O NPs.

	**OD values of groups**					
	**24 h**	**sd**	**48 h**	**sd**	**72 h**	**sd**
**Control**	0.1654	0.00462	0.1680	0.00430	0.2368	0.00409
**MAO**	0.1822	0.00593	0.2132	0.00926	0.2424	0.00871
**MAO-Ag1**	0.1804	0.00344	0.1674	0.00230	0.2546	0.00706
**MAO-Ag2**	0.1708	0.00370	0.1688	0.00239	0.2746	0.00751
**MAO-Ag3**	0.1706	0.00462	0.1688	0.00602	0.2398	0.00563
